# The involvement of prostaglandins in the contractile function of the aorta by aldosterone

**DOI:** 10.1186/1756-0500-4-125

**Published:** 2011-04-14

**Authors:** Danita Eatman, Katie Peagler, Jana Watson, Aisha Rollins-Hairston, Mohamed A Bayorh

**Affiliations:** 1 Department of Pharmacology/Toxicology, Morehouse School of Medicine, Atlanta, Georgia, USA

## Abstract

**Background:**

Aldosterone, one of the major culprits associated with the renin-angiotensin-aldosterone system (RAAS), is significantly elevated following high salt administration in Dahl rats. Since we have previously demonstrated that aldosterone (ALDO) upregulates cyclooxygenase (COX) expression in the kidney, the present study was design to assess whether prostaglandin release is involved in the effects of chronic aldosterone treatment on vascular function of the aorta from nonhypertensive Dahl salt-sensitive rats.

**Findings:**

The effects of aldosterone on arachidonic acid metabolism and on the expression of cyclooxygenase (COX)-2 were evaluated in the Dahl salt sensitive (DS) rat aorta, renal, femoral and carotid arteries. DS rats on a low salt (0.3% NaCl) diet were treated with or without ALDO for four weeks. Indirect blood pressure (BP), the release of prostacyclin (PGI_2_) and prostaglandin E_2_, and the expression of COX-2 were measured to assess the vascular remodelling by aldosterone. Vascular function was also assessed by contractile responsiveness in the aorta to phenylephrine. ALDO increased BP (17 ± 1%) and inhibited the basal release of PGE_2_. ALDO enhanced vascular reactivity to phenylephrine and up regulated the expression of COX-2 in both aorta and renal vessels but reduced COX-2 expression in the femoral artery.

**Conclusions:**

These data reveal that the effect of ALDO in the vasculature is tissue specific and may involve the inhibition of PGE_2 _release. Thus, suggesting a role for prostaglandins in the vasculopathic aspects of aldosterone.

## Background

Salt-induced hypertension associated end-organ damage may be due to direct effects of aldosterone on cardiac, renal and other vascular tissues [1,2]. We previously reported the salt content of the diet was crucial for the hypertensive effect of aldosterone [3]. In fact, aldosterone produces a time-dependent increase in blood pressure in the presence of a low-salt diet, but not in the presence of a high-salt diet, implicating an inappropriate control of plasma aldosterone levels during the development of aldosterone-mediated hypertension. Also, we have found that aldosterone-induced endothelial dysfunction in both normotensive and hypertensive rats may be related to an involvement of the prostanoid pathway. Others have shown that the effect of aldosterone in the vasculature was associated with an overproduction of prostacyclin [4,5].

Prostacyclin (PGI_2_) and prostaglandin E_2 _(PGE_2_) have been found to be predominant products of cyclooxgenase metabolism [6]. Reduced bioavailability, of one or all of these protective endothelial factors, results in vascular dysfunction, a condition that has been documented in patients with hypertension, diabetes, atherosclerosis, and angina [7-9]. Aldosterone modulates membrane receptors and signalling molecules and influences the actions of a variety of agents to sensitize the vasculature to effects of various agents that induce vasoconstriction or result in direct effects on growth and remodelling. However, the contribution of cyclooxgenase and its products to the vascular effects of aldosterone on specific blood vessels needs to be further examined. The renin-angiotensin-aldosterone system is involved in alterations of vascular function in hypertensive patients, the study of aldosterone effects on vascular function could be especially relevant in the hemodynamic abnormalities associated with hypertension. Thus, the present study was design to assess whether prostaglandin release is involved in the effects of chronic aldosterone treatment on vascular function of the aorta from nonhypertensive Dahl salt-sensitive rats.

## Methods

### Animals and diets

Male Dahl S (SS/Jr) rats (130-150 g, 4-5 weeks old, twelve rats) were obtained from Charles River Laboratories, Wilmington, MA. All protocols involving animals were previously approved by the Morehouse School of Medicine Animal Care Committee. Guidelines followed were those of the Public Health Service and the revised animal welfare act as regulated by USDA. The rats were housed in a room with 12 h light - dark cycles and, after one week of acclimation, the rats were divided into two groups (six rats/group) and food and water were available ad lib. Group 1 was fed a low salt (LS; 0.3% NaCl) and Group 2 was fed a LS with aldosterone (ALDO;0.05 mg/kg/day) for three weeks. The systolic blood pressure and body weight was measured prior to start of diet and weekly thereafter. The salt diet was obtained from Harlan Teklad, Madison, WI. Diets, blood collection, and blood pressure measurements have been previously described [10].

### Vascular Preparation

The rats were euthanized by rapid decapitation, in accordance with American Veterinary Medical Association and the MSM Laboratory Animal Care Committee guidelines. The thoracic aorta was rapidly but gently removed to avoid stretching or damaging the endothelium and was placed in cold (4°C), gassed (95%O_2_/5% CO_2_), Krebs-Henseleit-bicarbonate solution (KHB)as described previously[11]. The aorta was cleaned of all adipose and connective tissue, and the midthoracic region was cut into rings (3 mm long) for vascular reactivity and prostanoid release experiments or kept intact for western blot analysis.

For the vascular reactivity experiments, two adjacent aortic rings were prepared from each animal and were studied in a paired approach. The rings were mounted on two 25-gauge stainless steel wires; one was attached to a stationary stainless steel rod and micrometer and the other was attached to a force displacement transducer (model FT-03D, Grass) for measurement of isometric tension. The transducers were connected to DataTrak recording and analysis program (Global Scientific LLC) for a continuous record of contractile tension. Immediately after being mounted, the aortas were immersed in water-jacketed organ baths filled with 15.0 ml of KHB solution, maintained at 37°C, and continuously gassed with 95% O_2_-5% CO_2_. The aortic rings were gradually stretched (over a 30-min period) to an optimal passive tension of 2.50 g [11,12] and then equilibrated for 90 min. During the equilibration period, the KHB solution in the organ baths was replaced with fresh KHB solution every 20 min. The passive tension was adjusted to maintain 2.50 g throughout the equilibration and experimental periods. After the equilibration period, the aortic rings were stabilized by a single near-maximal contraction phenylephrine (PE; 1 × 10^-6 ^M) to avoid possible tachyphalaxis to PE during the subsequent cumulative concentration- response experiments. After each contraction reached a stable plateau tension, the endothelium-dependent vasodilator acetylcholine was added to the baths (1 × 10^-7 ^M) to assess functional integrity of the endothelium. The baths were rinsed twice and the aortas were allowed to reequilibrate in fresh KHB solution for 30-45 min before further experimentation. The effects of aldosterone on vascular reactivity to PE were examined by obtaining cumulative concentration responses to PE (10^-9^-10^-4 ^M) in endothelium-intact aortas.

For the *v*ascular prostanoid release experiments, the aortic rings (3 mm long) were prepared from intact thoracic aortas and were placed into chilled, gassed KHB solution and allowed to stabilize for at least 30 min. The rings were then transferred into 12 × 75-mm plastic culture tubes with 2 ml chilled KHB, and gradually warmed up to 37°C. After preincubation for 30 min at 37°C, the KHB solution was carefully aspirated, and then 1.0 ml of KHB was added to the tissues, gassed continuously, and incubated for 45 min at 37°C. After incubation, the KHB was collected and stored at - 80°C until assayed for 6-keto-prostaglandin F_1αalpha _(stable metabolite of prostacyclin) and prostaglandin E2 by enzyme immunoassay (Cayman Chem. Corp., Ann Arbor, MI) according to the manufacturer's instructions. Basal release reflected the steady- state release of the prostanoids into the incubation medium (KHB) during the 45-min incubation period and was normalized by dry weight of aortic rings and expressed as picogram/milligram dry tissue weight/45 min as described by Li and Stallone [13].

### Western Blot Analysis

Tissue samples of aorta, carotid and femoral were homogenized in ice cold T-PER lysis buffer containing protease inhibitor cocktail (Pierce, Rockford, IL). Homogenates were pellet at 3000 g for 5 minutes at 4°C and supernatants were collected for Western blotting. Protein concentrations were determined by Bradford protein assay (Bio-Rad, Hercules, CA). To detect protein levels of COX-2, tissue lysates (20 μg) were separated by 4-12% Bis-Tris gels (Invitrogen, Carlsbad, CA) and electro-transferred onto polyvinylidene fluoride (PVDF) membranes. The membranes were incubated overnight at 4°C with specific antibodies to COX-2 (Santa Cruz Biotechnology, Santa Cruz, CA). After incubation with the secondary antibody, immune complexes were detected using the enhanced chemiluminescence method. Proteins levels from western blots were evaluated by quantifying the band intensities using NIH Image version 1.6.3 software.

### Statistics

A mean ± SEM was calculated for each group. Statistical significance (*P *< 0.05) was evaluated by the Mann Whitney test (SigmaStat, SPSS, Inc., Chicago, IL).

## Results

**Figure 1:**
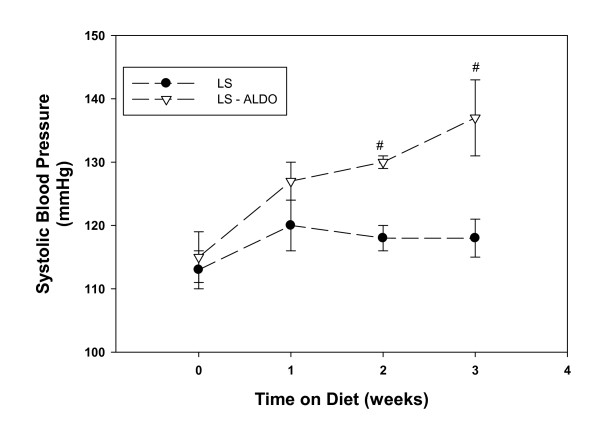
**Effect of ALDO on Systolic Blood Pressure**. Indirect blood pressure was measured prior to start of experiment and weekly thereafter in Dahl SS rats fed a low salt diet (0.3%) with or without aldosterone. Values are means ± SEM, n = 6. #P ≤ 0.05, LS vs. LS-ALDO.

**Figure 2:**
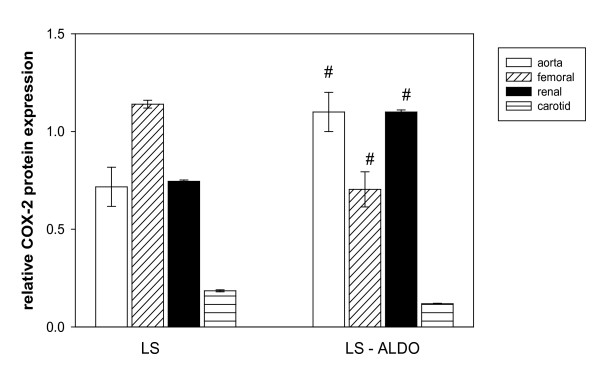
**Effect of aldosterone on COX-2 expression is tissue specific**. Dahl salt-sensitive (SS) were fed a low salt (0.3%, LS) diet with or without aldosterone (ALDO; LS-ALDO) diet for three weeks. Values are means ± SEM, n = 6. #P ≤ 0.05, LS vs. LS-ALDO.

**Figure 3:**
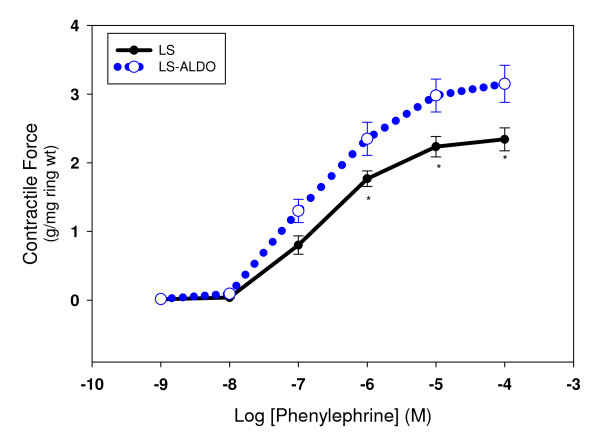
**Effect of chronic aldosterone treatment on phenylephrine-induced vasoconstriction in rat thoracic aortic rings**. The thoracic aortic rings from Dahl SS rat treated with or without ALDO for three weeks was constricted with phenylephrine (10^-9 ^- 10^-4 ^M). Isometric tension was recorded and normalized by dry weight of aortic rings and expressed as g/mg ring weight. Values are means ± SEM, n = 6. *P ≤ 0.05, LS vs. LS-ALDO.

Treatment with ALDO increased systolic blood pressure significantly from baseline to week 3 (Figure 1). The greatest increase in blood pressure by ALDO occurred at week 2 and 3. Body weight increased over time in both groups but was not significantly different (data not shown). To address the effect of aldosterone on vascular remodeling, endothelium-derived factors (i.e. prostacyclin, PGE_2_) were measured as well as changes in the vascular expression of COX-2. Basal release of prostacyclin from the aortic rings was similar between the groups; however, basal release of PGE_2 _was 62% higher in the control when compared to the ALDO treated group (Table 1). The expression of COX-2 protein was measured in various vessels from these animals to determine if the aldosterone mediated upregulation of COX-2 was tissue specific. In the control groups, COX-2 expression was highest in the femoral arteries when compared to the aorta, renal and carotid vessels (Figure 2). However, chronic treatment with ALDO produced a significant increase in COX-2 expression in the aorta and renal vessels and reduced expression in the femoral artery. Contractile response to phenylephrine was used to assess the effect of ALDO on vascular function. ALDO treatment increased the responsiveness of the aortic rings to phenylephrine at concentrations 10^-9 ^- 10^-4 ^M (Figure 3).

**Table 1:** Status at week 3 of Dahl salt-sensitive male rats fed a low salt (LS) diet with or without aldosterone (ALDO).

Parameter	LS	LS-ALDO
Body Weight	322 ± 6	315 ± 5
Prostacyclin	1877 ± 165	1636 ± 92
Prostaglandin E_2_	300 ± 43	115 ± 14^a, b^

Values are means SEM, n = 6; ^a^LS vs. LS-ALDO; prostacyclin vs. PGE_2_. Body weight is expressed in grams and prostaglandin values are expressed as pg/ml/mg ring wt

## Discussion

We sought to examine the impact of aldosterone on vascular remodeling and function by assessing changes in aortic contractility. The underlying molecular mechanisms of aldosterone on prostaglandins were also determined by measuring the expression of COX-2 and the release of prostaglandins. Aldosterone was given in the presence of low sodium diet to salt-sensitive rats. In previous studies, we have shown that when these rats are fed an enhanced sodium diet, angiotensin II and aldosterone levels are increased and prostaglandin levels are lowered [4]. Therefore, we hypothesized that activation of the mineralocorticoid receptor with aldosterone contributes to the vascular remodeling and dysfunction in salt-induced hypertensive rats and involves prostaglandin formation. The major findings of this study reveal that ALDO-mediated enhancement of contractile function in the rat aorta may be due in part to inhibition of PGE_2 _release. We also found that the response to aldosterone was tissue specific.

Based on the COX-2 protein expression in the aorta and renal vessels, we suspect that the prostanoids are highly involved in the vasculopathic effects of aldosterone. Several studies have demonstrated that aldosterone stimulates an increase in the activity of various inflammatory agents, including COX-2-derived products, in some experimental models of cardiovascular diseases [3,5]. COX-2 stimulates renin release [14], which plays a role in hypertension via the renin-angiotensin-aldosterone system. Excessive expression of COX-2 as observed in this study may stimulate renin-angiotensin-aldosterone system and as suggested by others lead to increased arterial pressure and vascular lesions in inflammatory diseases, including hypertension and diabetes [15,8]. We anticipated that an abundance of COX-2 would lead to an increase in prostanoid release since COX mediates the rate-limiting step in arachidonic acid metabolism. However, we found in the aorta that prostacyclin levels remained unchanged and PGE2 release was significantly diminished during aldosterone treatment. Prostaglandin levels are normalized by ring weight instead of protein levels and this might account for the unexpected decline in prostaglandin levels; however, others have used dry tissue weight to normalize prostanoid findings [13]. Besides, we have found that plasma levels of prostaglandins are also decreased by ALDO when COX-2 levels are elevated (data not published). We suspect that ALDO-mediated reduction in prostaglandin E_2 _release may reflect a decrease in PGE2 synthase levels in the rat aorta and thus lead to the decline in PGE_2 _production.

This decline in PGE_2 _might explain the enhanced responsiveness to phenylephrine observed in this study. Vascular function was assessed in the aorta following chronic treatment with ALDO. The heighten responsiveness to phenylephrine in the aortas from the ALDO-treated rats and the decrease in prostaglandin E_2 _release suggested an involvement of the prostaglandins in the contractile function mediated by aldosterone. Others have shown that chronic treatment with aldosterone impaired endothelial function in mesenteric resistance arteries under normotensive and hypertensive conditions by increasing COX-2-derived prostacyclin and thromboxane A_2 _[5]. However, in this study, COX-2-derived prostacyclin was unchanged in the aorta. Although the rat aorta is a large conduit vessel not involved in the regulation of peripheral resistance, it has been well established that the rat aorta is a relevant model for the study of vascular function in vitro, the aorta's sensitivity to vasoconstrictor and vasodilator agonists is often much lower than smaller resistance-level vessels; thus this might offer some insight into the observed differences between these vessels. Besides, in this study, COX-2 protein expression varied between blood vessels by up regulating expression in the aorta and renal arteries but down regulating expression in the femoral arteries. ALDO may enhance contractile responses in the rat aorta to phenylephrine by inhibiting the release of PGE_2 _and in the mesenteric arteries by increasing prostacyclin and thromboxane A2 release. In conclusion, these studies suggest a role for prostaglandins in the vasculopathic aspects of aldosterone.

## Competing interests

The authors declare that they have no competing interests.

## Authors' contributions

DE participated in the conception, design and coordination of the study and analyzed the data from the COX-2 and vascular reactivity studies and drafted the manuscript. KP carried out the prostanoid release studies and analysis. JW carried out the vascular reactivity studies. AR-H carried out the western blot analysis. MAB participated in the conception and design of the study and in the blood pressure measurements. All authors read and approved the final manuscript.
